# Isolation and Characterization of the Novel Bacteriophage AXL3 against *Stenotrophomonas maltophilia*

**DOI:** 10.3390/ijms21176338

**Published:** 2020-09-01

**Authors:** Jaclyn G. McCutcheon, Andrea Lin, Jonathan J. Dennis

**Affiliations:** Department of Biological Sciences, University of Alberta, Edmonton, AB T6G 2E9, Canada; jgmccutc@ualberta.ca (J.G.M.); alin3@ualberta.ca (A.L.)

**Keywords:** bacteriophage, phage therapy, *Stenotrophomonas*, phage genomics, phage receptor

## Abstract

The rapid increase in the number of worldwide human infections caused by the extremely antibiotic resistant bacterial pathogen *Stenotrophomonas maltophilia* is cause for concern. An alternative treatment solution in the post-antibiotic era is phage therapy, the use of bacteriophages to selectively kill bacterial pathogens. In this study, the novel bacteriophage AXL3 (vB_SmaS-AXL_3) was isolated from soil and characterized. Host range analysis using a panel of 29 clinical *S. maltophilia* isolates shows successful infection of five isolates and electron microscopy indicates that AXL3 is a member of the *Siphoviridae* family. Complete genome sequencing and analysis reveals a 47.5 kb genome predicted to encode 65 proteins. Functionality testing suggests AXL3 is a virulent phage and results show that AXL3 uses the type IV pilus, a virulence factor on the cell surface, as its receptor across its host range. This research identifies a novel virulent phage and characterization suggests that AXL3 is a promising phage therapy candidate, with future research examining modification through genetic engineering to broaden its host range.

## 1. Introduction

*Stenotrophomonas maltophilia* is an aerobic, Gram-negative bacterium that is ubiquitous in the environment, often found in close association with plants helping to promote plant growth and nutrient breakdown in the rhizosphere [1,2,3]. Unfortunately, this bacterium is rapidly rising in prevalence as a nosocomial and community-derived opportunistic pathogen, associated with a broad spectrum of clinical syndromes [4,5]. *S. maltophilia* is easily transmitted between patients and healthcare workers via direct contact and cough-generated aerosols and while predominantly associated with respiratory tract infections, this bacterium can also cause wound and soft tissue infections, urinary tract infections, severe bacteremia, meningitis, endocarditis, pneumonia, catheter-related bacteremia/septicemia and acute exacerbations in patients with cystic fibrosis [3,5,6]. Once an infection is established treatment is difficult due to the innate and adaptive antibiotic resistance mechanisms encoded by *S. maltophilia* that provide resistance to a broad range of antibiotics including trimethoprim-sulfamethoxazole, β-lactams, macrolides, cephalosporins, fluoroquinolones, aminoglycosides, carbapenems, chloramphenicol, tetracyclines, and polymyxins [3,5]. In addition to inducible chromosomally encoded β-lactamases, multidrug efflux pumps and low outer membrane permeability to defend against antibiotic treatments, *S. maltophilia* encode several virulence factors. These include extracellular enzymes such as proteases, esterases, lipases and haemolysin, and the cell-associated virulence factors lipopolysaccharide and type IV pili that contribute to antimicrobial resistant biofilm formation on abiotic surfaces and host tissues, which aid in the establishment of chronic infections [5]. The tenacity of this pathogen and its extreme multidrug resistance make typical antibiotic treatments ineffective against these infections, emphasizing the need for alternative treatments.

Phage therapy, the clinical application of bacteriophages for the treatment of bacterial infections, is one alternative treatment option gaining traction in North America [7,8,9]. Previously overlooked in the 1940’s with the advent of antibiotics in the West, phages are bacterial viruses that recognize and bind to their bacterial host via recognition of a specific cell surface receptor to infect and kill their target bacterial species. This makes phages incredibly specific to their host and in a therapeutic context, the application of phages targeting a specific bacterial pathogen will leave beneficial microbiota unharmed, unlike antibiotics. The use of phages for human therapy requires extensive phage characterization, including host range analysis, genomic characterization and identification of potential moron genes, lifestyle determination and identification of cell surface receptors. In the last two decades, numerous phages against *S. maltophilia* have been isolated and characterized to various extents for their therapeutic potential [10,11,12,13,14,15,16,17,18,19,20,21,22,23,24]. Herein we describe the isolation and characterization of a novel *S. maltophilia* phage, AXL3, unrelated to any phages currently sequenced in the NCBI database. We show that this phage recognizes and binds to the major pilin subunit PilA of the type IV pilus, a virulence factor of many bacterial pathogens, and has potential as a phage therapy candidate against multidrug resistant *S. maltophilia*.

## 2. Results and Discussion

### 2.1. Isolation, Morphology, and Host Range

Bacteriophage AXL3 (vB_SmaS-AXL_3) was isolated using clinical *S. maltophilia* strain D1585 from soil collected at the Patrick Seymour Alpine Garden at the University of Alberta Botanic Gardens. *S. maltophilia* phages have previously been isolated from soil and rhizosphere samples [10,11,17,18,19]. This phage forms small plaques 0.78 ± 0.12 mm in diameter with clear borders after 16 h incubation on its main host, D1585.

**Figure 1:**
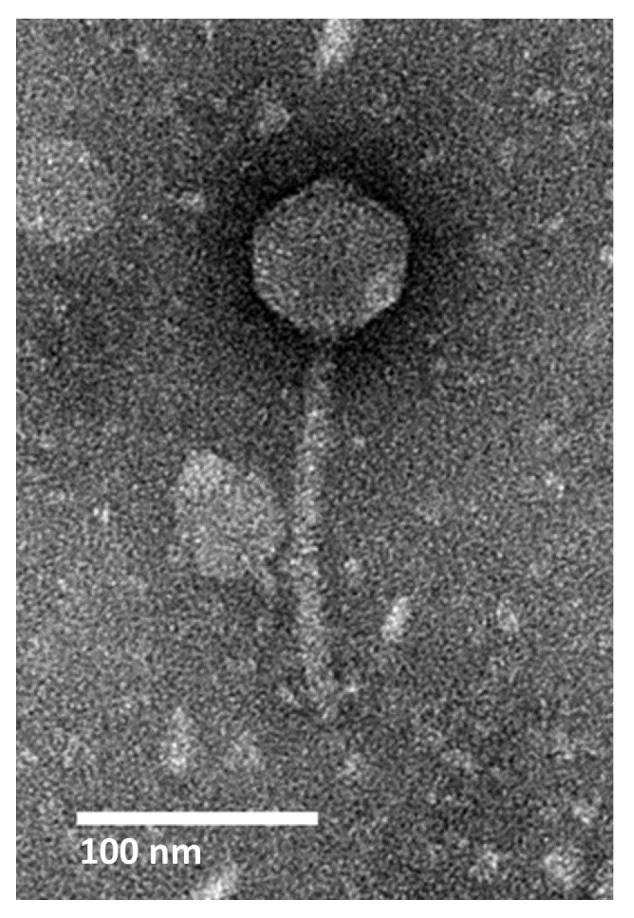
Transmission electron micrograph of AXL3. High titer lysate was stained with 4% uranyl acetate on a copper grid and viewed at 110,000× magnification with a transmission electron microscope. Measurements of 10 phage particles have an average capsid length and width of 64 nm and 63 nm, and tail length of 145 nm.

Transmission electron microscopy (TEM) classifies AXL3 as a *Siphoviridae* phage having a B1 morphotype [25] based on the long, noncontractile tail averaging 145.3 ± 5.4 nm in length and isometric head with an average capsid length and width of 64.3 ± 3.2 nm and 63.3 ± 4.3 nm, respectively (Figure 1). No tail fibers were observed in the TEM images.

Host range analysis using a panel of 29 phenotypically distinct *S. maltophilia* clinical isolates reveals a narrow tropism, with AXL3 capable of infecting only 5 strains, D1585, 213, 280, D1576, and D1568, and propagating to a high titre of 10^10^ PFU/mL on strain D1585 (Table 1). Although the original AXL3 lysate that was propagated on strain D1585 did not infect strains 280 and D1576 at high efficiency, successive passaging of AXL3 on these strains produced lysates with higher efficiencies of plating, forming plaques on 280 when diluted to 10^−5^ and clearing on D1576 when diluted to 10^−2^, indicating that AXL3 successfully replicates in these hosts. Extended host range analysis using a panel of 26 *Pseudomonas aeruginosa* strains did not yield successful infections (data not shown), unlike the broad host range *S. maltophilia* phages DLP1 and DLP2 [10]. Analysis of AXL3-resistant D1585 and 213 single colony isolates by PCR with internal AXL3-specific primers produced a low number of isolates positive for AXL3 gDNA. These isolates were PCR-negative after passaging twice, indicating that AXL3 cannot stably lysogenize its host, however pseudolysogeny may be possible. A one-step growth curve on strain D1585 shows a long productive cycle for AXL3, having a latent period of approximately 2.5 h, and a burst size of approximately 38 virions per cell at 6.5 h (Figure S1).

### 2.2. Receptor Identification

**Figure 2:**
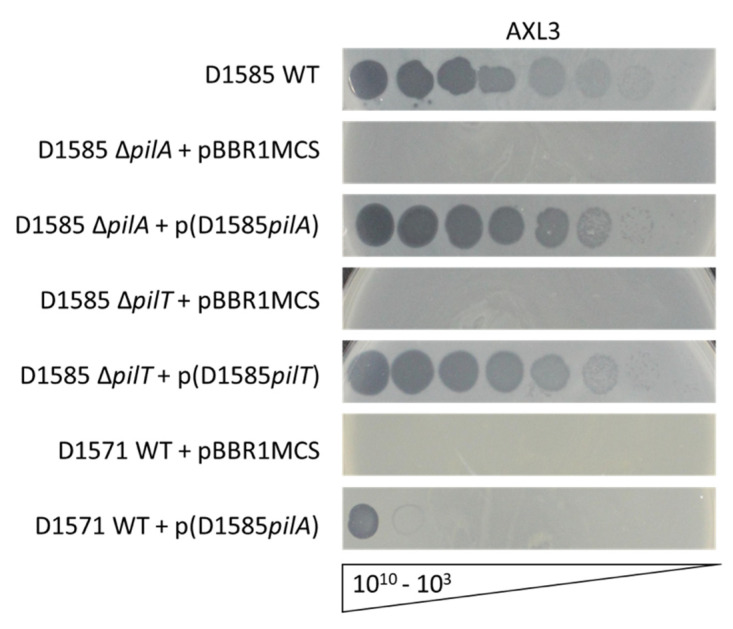
*S. maltophilia* bacteriophage AXL3 requires a functional type IV pili for infection. Wildtype (WT) *S. maltophilia* strain D1585 is susceptible to AXL3. Deletion of the major pilin subunit encoded by *pilA*, or the retraction ATPase encoded by *pilT*, abolishes infection by AXL3. Complementation restores phage infection to wildtype levels. Exogenous expression of the D1585 *pilA* gene in a phage-resistant host, D1571, permits AXL3 infection. Images are representative of three biological replicates, each with three technical replicates.

Four *Siphoviridae* phages previously isolated from soil samples that are capable of infecting *S. maltophilia* strain D1585 were found to bind the type IV pilus as their cell surface receptor [18,19,26]. The type IV pilus is a virulence factor on the surface of many Gram-negative bacterial pathogens and is used by the cell to adhere to surfaces, form biofilms, and migrate along a surface, a form of motility known as twitching [27]. To determine if the type IV pilus is also involved in AXL3 adhesion, previously constructed D1585 pilus mutant strains [19,26] were tested in a plaque assay using serially diluted phage stock. *S. maltophilia* D1585 Δ*pilA* lacking the major pilin subunit, and therefore lacking an external pilus, is completely resistant to AXL3 infection, showing an absence of cell lysis (Figure 2). Phage infection is restored to wildtype levels upon complementation with the *pilA* gene on a plasmid, plaquing at 10^3^ PFU/mL. Additionally, a hyper-piliated, non-motile D1585 Δ*pilT* mutant lacking a retraction ATPase required for pilus depolymerization was also resistant to AXL3 infection. This mutant is capable of assembling type IV pili on its cell surface however the pili are non-functional and unable to retract. This strain grows poorly in liquid, as observed in the speckled lawn, however this phenotype and susceptibility to AXL3 are restored to wildtype levels by complementation with the *pilT* gene (Figure 2).

Unlike the well-characterized *P. aeruginosa* type IV pili system that encodes a single major pilin protein, *S. maltophilia* encodes two major pilin homologs in tandem, *pilA1* and *pilA2*, similar to many *Xanthomonas* species within the *Xanthomonadaceae* family [28]. This is true of strain D1585 in the present study, which carries two major pilin genes directly upstream of *pilB* that encodes the assembly ATPase and clustered within genes encoding the platform protein, PilC, prepilin peptidase, PilD, and two-component system regulatory proteins PilS and PilR. This organization is present in other *Stenotrophomonas* and *Xanthomonas* species, however the function of these duplicated major pilins compared to the canonical system is unknown [28]. In the above D1585 *pilA* mutant, we deleted the *pilA1* paralog leaving *pilA2* intact directly downstream. Deletion of the single gene abolishes infection by AXL3, as observed in Figure 2, as well as infection by *S. maltophilia* phages DLP1, DLP2, DLP3, and DLP4 [18,19,26], and was previously observed to abolish pili function investigated via loss of twitching motility [26]. These results suggest that the major pilin PilA1 is required for expression of a function pili in *S. maltophilia* while the role of PilA2 is unknown. Because deletion of *pilA1* was sufficient to disrupt phage infection we did not explore mutagenesis of *pilA2* further in this study, however the presence of two neighboring *pilA* genes with a pairwise identity of 51.1% across the length of the protein suggests that *S. maltophilia* may be capable of antigenic variation of its type IV pilus to evade host immune systems and alter pili function, as observed in *Neisseria* species [29].

To further test type IV pili recognition by AXL3 for host infection, we expressed the D1585 *pilA1* gene on a plasmid in an AXL3-resistant strain, D1571, and examined phage susceptibility. Although pilin subunits are highly variable between species, and even strains, the type IV pilus assembly machinery is highly conserved and allows the expression of exogenous pilins and assembly of functional heterogenous pili [26,30]. Remarkably, the D1571 strain expressing the D1585 *pilA* gene is susceptible to AXL3 infection, showing complete lysis of the bacterial lawn at 10^10^ PFU/mL and partial infection at 10^9^ PFU/mL (Figure 2). No evidence of phage infection appears in the D1571 empty vector control. Although infection efficiency is low, repeat passaging of AXL3 on this variant produces lysate with a titre of 10^10^ PFU/mL and forms plaques at a 10^−7^ dilution after three infection cycles. This was not observed on the D1571 wildtype strain, indicating that the D1585 pilin is sufficient for infection and phage replication. Together these data show that phage AXL3 uses the type IV pilus as its cell surface receptor, is capable of recognizing and binding directly to the PilA subunit and requires a functional pilus to reach the cell surface for successful infection.

AXL3 is the fifth documented *S. maltophilia* phage to use the type IV pilus as its receptor, with all five phages isolated from soil [10,18,19,26]. It is possible that the competitive advantage of type IV pili to aid in the colonization of plants [2,31] has selected for the use of these structures as phage receptors in soil microbes. It is unknown if the type IV pilus is a favoured receptor of *S. maltophilia* phages isolated from other environmental sources, such as water and sewage, as the receptors for these phages have not been examined [12,13,14,15,16,22,23,24,32]. However, the T4-like virulent phage Smp14 has been observed to bind to the poles of *S. maltophilia* cells by TEM [21] where type IV pili are normally expressed. As type IV pili are also important for the colonization of medical devices and patients, phages that use pili as receptors are good candidates for an anti-virulence phage therapy strategy; should bacteria become resistant to phages through modification or loss of the type IV pili receptor, this mutation provides phage resistance at the cost of lowered virulence and reduced fitness compared to non-resistant cells [26,33].

### 2.3. Genomic Characterization

**Figure 3:**
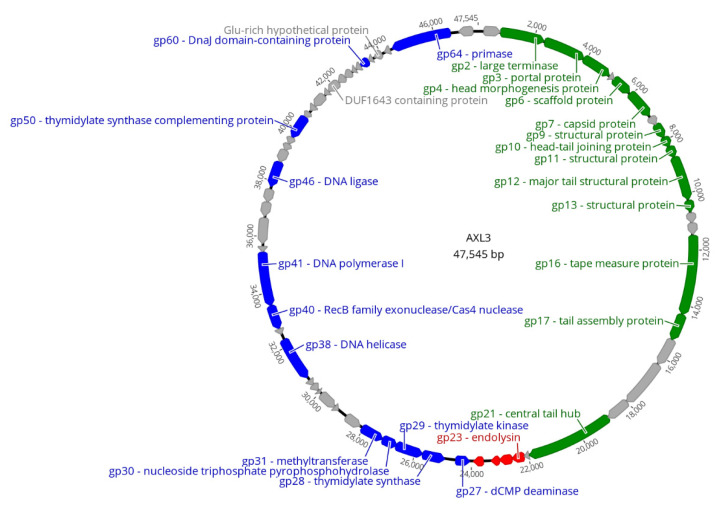
Circularized genomic map of AXL3. Scale (in bp) is shown on the outer periphery. Assigned putative functions for each of the 65 predicted open reading frames are as follows: lysis (red), DNA replication and repair (blue), virion morphogenesis (green), hypothetical (grey). No tRNA or lysogeny genes were identified. AXL3 has a GC content of 63.3%. Image created using Geneious Prime [36].

The AXL3 genome is 47,545 bp in length (Figure 3) with a GC content of 63.3%, which is slightly lower than the D1585 host GC content of approximately 67%. Interestingly, BLASTn analysis of AXL3 shows limited identity to other phages in the NCBI database, exhibiting a maximum identity of 67.65% with the *Siphoviridae Pseudomonas aeruginosa* phage JG012 over 4% of the AXL3 genome. This region of identity aligns the AXL3 region containing genes AXL3_12 and AXL3_13 with JG012 major tail structural proteins encoded by genes 13 and 14. BLASTn analysis of the AXL3 genome against *Stenotrophomonas* sp. (taxid:40323) produced no significant results with greater than 2% query coverage, indicating that remnants of this phage are not present as prophage elements in any *Stenotrophomonas* species sequenced and further supports the virulent nature of this phage.

Restriction fragment length polymorphism (RFLP) analysis of purified AXL3 gDNA using 37 restriction endonucleases with recognition sequences present in the genome showed successful digestion with only two enzymes, *TasI* and *Tru1I* (Figure S2). These enzymes contain only A/T bases in their recognition sequences. This suggests that the AXL3 genome is modified or contains atypical bases to protect it against host restriction-modification systems. Although the specific modifications are unknown, the 35 enzymes tested that could not digest the DNA contain G/C bases in their recognition sites, suggesting that these nucleotides may be altered in AXL3 gDNA to resist digestion. Phage genome resistance to restriction digestion is common and has been documented in other *S. maltophilia* phages to varying degrees [11,17,19,20,22], with phage DLP4 gDNA resistant to a similar panel of enzymes as AXL3 [18].

AXL3 is predicted to encode 65 putative protein-coding genes (Table 2, Figure 3), producing a coding density of approximately 94%. The majority of start codons are ATG (56 of 65), with fewer GTG (8) present, and only gp14 using TTG. Most stop codons are TGA (43 of 65), with the remaining mainly TAA (20) and only two, gp27 and gp628, using TAG. No tRNA genes were identified. Functional predictions for the 65 putative proteins by BLASTp analysis produced significant matches for 43 proteins, with conserved domains identified in 22 of the proteins. Of the 43 proteins with hits, only 26 were given putative functions. The remaining 22 proteins did not have any identity to proteins in the NCBI database and were annotated as hypothetical (Table 2). While these hypothetical proteins are distributed throughout the AXL3 genome, a clear modular organization is evident, consisting of genes involved in DNA repair and replication (blue) on the negative strand and genes required for virion morphogenesis (green) and lysis (red) on the positive strand (Figure 3). The genome sequence of AXL3 with putative annotations has been deposited in Genbank with the accession number MT536174.

Based on the absence of nucleotide identity with known phages, it is possible AXL3 belongs to a new genus of phages. Given the mosaic nature of phage genomes and high degree of horizontal gene transfer between phages [34], we analyzed taxonomic relationships using vConTACT2 (v0.9.16), a network-based tool that uses phage genome protein content for viral classification and accurate clustering of phages at the genus level [35]. Analysis against the Prokaryotic Viral RefSeq94-Merged database classifies AXL3 as an outlier genome, meaning it is weakly connected with a cluster of sequences based on shared genes but lacks statistical significance to be included with the cluster. Visualization of the network shows that AXL3 shares similarities to 24 phage genomes that belong to three viral clusters or are also outliers (Figure S3). Further sampling of the virosphere is needed to strengthen the connection of AXL3 with existing taxonomy.

#### 2.3.1. DNA Replication and Repair Module

AXL3 encodes at least 12 genes related to DNA replication, repair and the generation and processing of nucleotides within the region AXL3_27 to AXL3_65 (Table 2, Figure 3). Within this co-directionally oriented gene cluster, gene products that could be assigned putative enzymatic functions by homology include dCMP deaminase (gp27), thymidylate synthase (gp28), thymidylate kinase (gp29), nucleoside triphosphate pyrophosphohydrolase (gp30), methyltransferase (gp31), DNA helicase (gp38), RecB family exonuclease (gp40), DNA polymerase I (gp41), DNA ligase (gp46), thymidylate synthase complementing protein (gp50), DnaJ molecular chaperone (gp60) and primase (gp64). An interesting hypothetical protein within this module is gp62, a glutamic acid-rich protein with 36 glutamic acid residues out of 99 in the protein, including a 27 glutamic acid repeat at its C-terminus. While polyamino acid repeats appear to be rare in prokaryotes and viruses [37], in eukaryotes, many proteins containing aspartic acid and glutamic acid-rich repeats are related to DNA/RNA functions [38].

A cluster of genes within this module encode enzymes involved in the thymidylate synthesis pathway, functioning to create dTDP from dCMP precursor. AXL3 encodes a putative deoxycytidylate (dCMP) deaminase (gp27) that processes dCMP to produce deoxyuridine monophosphate (dUMP) [39]. This product is the nucleotide substrate for thymidylate synthase, gp28, which catalyzes the conversion of dUMP into deoxythymidine monophosphate (dTMP) by means of reductive methylation using the cofactor 5,10-methylenetetrahydrofolate (CH_2_H_4_folate) [40]. Interestingly, AXL3 also encodes a putative thymidylate synthase complementing protein (gp50) that is typically found in organisms that lack a thymidylate synthase and complements its activity to convert dUMP into dTMP using FAD as an additional cofactor with CH_2_H_4_folate [41]. The dTMP product can be further processed by the AXL3 encoded thymidylate kinase (gp29) into dTDP on its way to being used in DNA synthesis. The identification of some proteins known to be involved in nucleotide biosynthesis and the restriction-resistant nature of the AXL3 gDNA suggest that the large number of hypothetical proteins in this module may be involved in the synthesis and incorporation of altered nucleotides, however further study is needed.

Of the 39 genes in this area, 11 gene products share high sequence identity with bacteria of the *Nitrospira* genus when BLASTp searches are limited to Bacteria (taxid:2) (Supplementary Materials Table S1). These Gram-negative, nitrite-oxidizing bacteria are widespread in the environment, found in both aquatic and terrestrial habitats, and play a key role in nitrogen cycling [42]. Specifically, ten proteins have top hits to the *Nitrospira* cf. *moscoviensis* strain SBR1015, including gp27 to gp31 described above. However, no AXL3 phage morphogenesis or lysis proteins had BLASTp hits to bacteria in this genus. The clustering of these genes, the significant sequence identity, and the location of these genes amongst non-prophage genes on the *Nitrospira* genome contigs, suggest that these AXL3 genes are of bacterial origin.

Of particular interest in this module is the identification of a Cas4 conserved domain in gp40 (Table 3). Cas4 proteins are DNA nucleases with 5′-3′ exonuclease activity shown to create recombinogenic ends for spacer acquisition in host CRISPR arrays to generate host immunity to invading DNA, including viruses and plasmids [43,44]. Phylogenetic analyses have identified *cas4* genes in many mobile genetic elements lacking CRISPR-Cas systems, including archaeal viruses and phages, suggesting the involvement of Cas4 nucleases in anti-defense functions [45]. In *Campylobacter jejuni* phages specifically, phage-encoded Cas4-like proteins have been identified and experimentally determined to be capable of incorporating host-derived spacers into the CRISPR array of their host bacterium during infection to evade host immunity [44]. BLASTp analysis reveals that AXL3 gp40 is conserved with these *Campylobacter* phage-encoded Cas4 proteins, suggesting a similar function of spacer acquisition in AXL3, however CRISPR-Cas immunity has not yet been characterized in *S. maltophilia* [18,46]. It is possible that phage-encoded Cas4-like proteins may play an uncharacterized role in defense against host restriction-modification systems; the *Campylobacter* phages encoding Cas4 nuclease homologs are also predicted to contain modified guanosine nucleotides that provide resistance to gDNA digestion with restriction enzymes [47], as observed in AXL3. A Cas4-like nuclease has also been identified in close proximity to deoxyarchaeosine (dG+) synthesis genes in the restriction-resistant genome of *E. coli* phage 9 g, suggesting a possible role for Cas4 in a restriction system of unmodified DNA, or degradation of host DNA for nucleotide recycling [48]. Further characterization of these phage-encoded Cas4 proteins will likely reveal uncharacterized defense and anti-defense systems.

#### 2.3.2. Virion Morphogenesis Module

The virion morphogenesis module of AXL3 consists of 22 genes (AXL3_1 to AXL3_22) oriented on the positive strand, the gene products for 13 of which were assigned functions based on BLASTp sequence identity (Table 2, Figure 3). Proteins involved in capsid assembly and packaging include the large terminase protein (gp2), portal protein (gp3), head morphogenesis protein (gp4), scaffold protein (gp6), and capsid protein (gp7). Eight proteins were identified as structural proteins involved in tail morphogenesis and phage assembly including three putative virion structural proteins (gp9, gp11, and gp13), a head-tail joining protein (gp10), a major tail protein (gp12), tape measure protein (gp16), tail assembly protein (gp17) and central tail hub protein (gp21). These proteins have sequence identity with phages specific to numerous bacterial species, including *P. aeruginosa, S. maltophilia*, and *Burkholderia cenocepacia.* This module follows the typical gene architecture for *Siphoviridae* morphogenesis modules; the capsid assembly genes are located upstream of the tail assembly genes that are organized starting with genes encoding the major tail proteins [49]. The tape measure protein generally corresponds to the length of the phage tail in *Siphoviridae* phages and is therefore the largest gene, however in AXL3 this gene is second in length to the gene encoding the central tail hub.

A conserved domain search revealed two domains of interest in the central tail hub protein, gp21, of AXL3 (Table 3). The pfam13550 Phage-tail_3 domain present in gp21 has been found in the tail proteins of other *S. maltophilia* phages experimentally confirmed to use the type IV pilus as a cell surface receptor [10,18,19,26]. We previously described the prediction that tail fibreless phages with baseplate proteins containing the Phage-tail_3 domain are capable of using the type IV pilus as a primary receptor [26], and the identification of this domain in the AXL3 central tail hub and functional analysis of the type IV pilus as the AXL3 receptor supports this hypothesis. Additionally, a Laminin G domain is present in gp21. Peters et al. [19] identified this domain in the tail proteins of two *Delepquintavirus* phages against *S. maltophilia* and predicted that they may play role in host specificity due to their variation in protein sequence, showing high pairwise identity at the C-terminus and low percent identity at the N-terminus; phage DLP3 was shown to bind the type IV pilus while the receptor for DLP5 is unknown [17]. This pattern of sequence variation is also observed in the tail fiber proteins for type IV pili-binding *Xylella* phages, Salvo and Sano [50]. Further experimental investigation into the function of gp21 in AXL3 as a receptor binding protein is currently underway to determine whether this protein plays a role in host recognition.

#### 2.3.3. Lysis Module

The lysis module directly follows the virion morphogenesis module in the AXL3 genome and consists of four genes (AXL3_23 to AXL3_26) (Table 2, Figure 3). The first gene in this module encodes a predicted endolysin, gp23, and has a conserved L-alanyl-D-glutamate peptidase domain identified by CD-Search (Table 3). This domain is found in other bacteriophage endolysins, including *Escherichia coli* T5 phage endolysin and the endolysins of *Listeria monocytogenes* phages A118 and A500, Ply118 and Ply500, respectively [51,52]. These cell wall lytic enzymes cleave between the l-alanine and d-glutamate residues of the peptidoglycan wall to cause cell lysis late in phage infection.

The genes downstream of the AXL3 endolysin are annotated as hypotheticals based on lack of sequence identity to known proteins in the NCBI database. Analysis of the gene products with TMHMM revealed the predicted presence of four transmembrane domains in gp24 and three transmembrane domains in gp25, suggesting potential functions as holin or i-spanin proteins [53]. Canonical holin proteins reside in the cytoplasmic membrane and upon triggering, create pores in the membrane to release phage endolysin into the periplasm to degrade the cell wall peptidoglycan. For complete cell lysis, disruption of the outer membrane is required by the spanin complex that consists of two proteins localized to the inner membrane and outer membranes [53]. No transmembrane domains were predicted in gp26, however analysis with LipoP 1.0 predicted gp26 to be a lipoprotein signal peptide with a predicted signal peptidase II cleavage site between amino acids 17 and 18. This suggests that gp26 acts as an o-spanin, anchored in the outer membrane and spanning the periplasm to reach the cytoplasmic membrane i-spanin protein [53].

## 3. Conclusions

These results characterize a novel virulent phage that is active against the multidrug resistant bacterial pathogen *S. maltophilia*. Genomic characterization of AXL3 reveals a 47,545 bp genome that is resistant to digestion with restriction enzymes containing G/C bases in their recognition sequences and predicted to encode 65 proteins, many of which have hypothetical functions. Phage AXL3 encodes numerous nucleotide processing enzymes and a putative Cas4 nuclease that may function to provide defense against host anti-phage defenses, however further experimentation is required. This phage is capable of infecting a narrow range of *S. maltophilia* hosts using the type IV pilus, an important virulence factor used for biofilm formation, adherence, and twitching motility [27]. Our results show that AXL3 interacts directly with the PilA subunit for host recognition and relies on host cellular retraction of the type IV pilus to reach the cell surface for successful infection. This is the fifth documented type IV pili-binding *S. maltophilia* phage [18,19,26]. No lysogeny genes were identified in our bioinformatic analyses and D1585::AXL3 or 213::AXL3 lysogens could not be isolated, indicating that AXL3 is a virulent phage. Further investigation of the receptor binding proteins for *S. maltophilia* type IV pili binding phages with vastly different host ranges may allow for genetic engineering of AXL3 to broaden its host range and increase its value as an “anti-virulence” candidate for phage therapy.

## 4. Materials and Methods

### 4.1. Bacterial Strains and Growth Conditions

Bacterial strains and plasmids used in this study are listed in Table 1 and Table 4. 29 phenotypically distinct *S. maltophilia* strains were used for host range analysis. Five *S. maltophilia* strains were acquired from the Canadian *Burkholderia cepacia* complex Research and Referral Repository (CBCCRRR; Vancouver, BC, Canada), with strain D1585 used for isolation of phages from soil samples. An additional 22 *S. maltophilia* strains were gifted from the Provincial Laboratory for Public Health–North (Microbiology), Alberta Health Services, and strains ATCC13637 and SMDP92 were gifted from Dr. Jorge Girón at the University of Virginia School of Medicine. All strains were grown aerobically overnight at 30 °C on half-strength Lennox (½ LB; 10 g/L tryptone, 5 g/L yeast extract, 5 g/L NaCl) solid medium or in ½ LB broth with shaking at 225 RPM. Media was supplemented with 35 µg/mL chloramphenicol (Cm) antibiotic for plasmid maintenance when necessary.

### 4.2. Phage Isolation, Propagation, and Host Range

AXL3 was isolated from soil collected in the Patrick Seymour Alpine Garden at the University of Alberta Botanic Gardens in Spruce Grove, Alberta, Canada using strain D1585 and a previously described extraction protocol [10]. Briefly, soil was incubated overnight with shaking at 30 °C in ½ LB broth, modified suspension medium (SM) (50 mM Tris–HCl [pH 7.5], 100 mM NaCl, 10 mM MgSO_4_), and *S. maltophilia* D1585 liquid overnight culture. Solids were pelleted by centrifugation and the supernatant was filter sterilized using a Millex-HA 0.45 µM syringe-driven filter unit (Millipore, Billerica, MA, USA) before using in a soft agar overlay with strain D1585. After overnight incubation, a single plaque was isolated using a sterile Pasteur pipette and suspended in 500 µL of SM with 20 µL chloroform to generate an AXL3 stock.

Propagation of AXL3 was performed using soft agar overlays as previously described [10,55]. Briefly, 100 µL of D1585 overnight culture was incubated with 100 µL of phage for 20 min, mixed with 3 mL of 0.7% ½ LB top agar, and overlaid onto ½ LB solid media. Plates were incubated at 30 °C overnight and those with confluent lysis were used to make high titre stocks by overlaying with 3 mL of SM, collecting the top agar and incubating with 20 µL chloroform per plate for 30 min at room temperature on a platform rocker. The supernatant was collected and filter sterilized as above and stored at 4 °C. The phage stock titre was determined using serial dilutions of phage stock into SM, followed by the soft agar overlay technique on strain D1585. Plaques were backlit and viewed under the magnifying glass of a New Brunswick Scientific colony counter (model C110) and plaque size was measured using digital calipers manufactured by Tresna (Guilin, China) and reported as the average from 10 plaques ± standard deviation.

Host range analysis was performed using a panel of 29 clinical *S. maltophilia* and 26 *Pseudomonas aeruginosa* strains. Soft agar overlays containing 100 µL liquid culture solidified at room temperature were spotted with 5 µL of a 10^10^ pfu/mL AXL3 stock at multiple dilutions and assayed for clearing and/or plaque formation after incubation at 30 °C for 24 h and 48 h. To assess phage replication in strains with low efficiency of plating, AXL3 was passaged on these strains using the soft agar overlays with 100 µL of a 10^−1^ diluted overnight culture and 300 µL AXL3 phage stock. Dilutions of the passaged lysates were spotted on overlays containing the strain of interest as described above to assess changes in efficiency of plating.

### 4.3. Electron Microscopy

For electron microscopy, phage stocks were prepared as above with the following modifications; ½ LB agarose plates and ½ LB soft agarose were used for overlays and a 0.22 µm filter was used for syringe-driven filtration. To visualize phages, a carbon-coated copper grid was incubated with 10 µL of phage lysate for 2 min and stained with 4% uranyl acetate for 30 s Transmission electron micrographs were captured using a Philips/FEI Morgagni transmission electron microscope with charge-coupled device camera at 80 kV (University of Alberta Department of Biological Sciences Advanced Microscopy Facility). The average capsid and tail dimensions ± standard deviation was calculated using Microsoft Excel based on measurements from 10 individual virions taken using ImageJ software (NIH, Bethesda, MD, USA) [56].

### 4.4. Determining Phage Lifecycle

Top agar overlay plates showing confluent lysis of D1585 by AXL3 were used to obtain phage resistant colonies for analysis. Briefly, 2 mL ½ LB broth was added to the plates and rocked at room temperature for 10 min. The broth was collected into microcentrifuge tubes and centrifuged at 5000× *g* for 5 min. The supernatant was discarded and 1 mL of fresh ½ LB was added to resuspend the pellet, followed by centrifugation. This wash step was repeated three times in total to remove contaminating phage. After the final centrifugation step and removal of the supernatant, the pellet was used to streak for isolation on ½ LB agar and incubated at 30 °C for 36 h. Single colony isolates were selected for further study and tested for superinfection resistance using overnight cultures of every isolate in a top agar overlay lawn spotted with 5 µL AXL3 phage. After 16 h incubation at 30 °C, the plates were examined and isolates without plaques or clearing in the phage spot were analyzed by colony PCR with TopTaq DNA Polymerase (Qiagen, Inc., Germantown, MD, USA) following manufacturer protocols using primers specific to AXL3 gDNA (F 5′-GTCAACGAGGAATCCAAGCC-3′; R 5′-CGAAGTGGTTGATCTGCTCG-3′).

### 4.5. One-Step Growth Curve

One-step analysis of phage growth on *S. maltophilia* D1585 was conducted to determine the burst size and latent period of AXL3 as previously described, with modifications [57]. Overnight liquid cultures of D1585 were subcultured and grown to an OD_600_ of 0.2 in full-strength LB at 30 °C. AXL3 lysate was added at an MOI of ~3 and allowed to adsorb for 5 min at room temperature, followed by incubation at 30 °C with aeration at 225 RPM. Samples were taken at 30 min intervals and immediately serially diluted in SM. Phage titres were determined by spotting 5 µL of each dilution on soft agar overlays containing D1585 culture. Resulting data from four replicates were analyzed using GraphPad Prism 8 (GraphPad Software Inc., San Diego, CA, USA).

### 4.6. Phage Plaquing Assays

AXL3 plaquing ability was determined by spotting on bacterial soft agar overlays, as previously described [26]. Briefly, 100 µL of overnight culture was mixed with 3 mL of 0.7% ½ LB top agar, overlaid onto ½ LB agar with or without 35 µg/mL Cm and allowed to dry at room temperature for 30 min. AXL3 phage stock was standardized to 10^10^ PFU/mL on *S. maltophilia* D1585 and tenfold serially diluted in SM to 10^3^ PFU/mL. 5 µL of each dilution was spotted onto the prepared plates and incubated at 30 °C for 18 h. Efficiency of plating on each strain was repeated in biological and technical triplicate.

### 4.7. Phage DNA Isolation, RFLP Analysis, and Sequencing

AXL3 genomic DNA (gDNA) was isolated from bacteriophage lysate by phenol/chloroform extraction and ethanol precipitation. A 1 mL aliquot of high titre filter sterilized phage lysate was nuclease treated with 1 µL of 10 mg/mL DNase I (Thermo Scientific, Waltham, MA), 10 µL 100× DNase I buffer (1 M Tris-HCl, 0.25 M MgCl_2_, 10 mM CaCl_2_), and 0.6 µL of 10 mg/mL RNase A (Thermo Scientific) and incubated at 37 °C for 1 h to degrade contaminating bacterial nucleic acids. Following incubation, 40 µL of 0.5 M EDTA was added to inactivate DNase I and 2.5 µL of 25 mg/mL Proteinase K (Applied Biosystems, Carlsbad, CA) and 50 µL 10% SDS was added and incubated at 55 °C for 1 h to release phage DNA. The treated lysate was split into two 1.5 mL microcentrifuge tubes and mixed with an equal volume of a 1:1 phenol:chloroform mixture followed by centrifugation at 17,900× *g* for 5 min. The aqueous layers were transferred to new tubes and phenol:chloroform extracted twice more followed by a single chloroform wash to remove residual phenol. Phage gDNA was precipitated from each aqueous layer by the addition of 1 mL ice-cold 95% EtOH and 50 µL 3M sodium acetate and incubated on ice for 30 min followed by centrifugation at 17,900× *g* for 10 min. The pellet was washed with 1 mL ice-cold 70% EtOH and the supernatant was removed following centrifugation. The gDNA pellet was air dried and dissolved in 50 µL sterile milli-Q water overnight. A NanoDrop ND-1000 spectrophotometer (Thermo Scientific, Waltham, MA) was used to determine purity and concentration of phage gDNA.

Restriction fragment length polymorphism (RFLP) analysis was used with 32 FastDigest (Thermo Scientific) restriction enzymes (AccI, MspI, HpaII, HhaI, Bsh1236I, MauBI, PdmI, HaeIII, NheI, AciI, Eam1105I, SmaI, XbaI, BamHI, KpnI, ApaI, SacI, EcoRI, HindIII, SalI, PstI, ClaI, XhoI, NotI, StuI, BglII, AvrII, PvuI, MscI, StyI, TasI, Tru1I) and five High-Fidelity (New England Biolabs) restriction enzymes (BsaAI, PspXI, EcoO109I, BsaI, AlwNI). Restriction digest reactions were set up using 1 µL of enzyme, 2 µL of restriction buffer, 1 µg of AXL3 DNA and topped up to 20 µL with sterile milliQ water. Reactions were incubated at 37 °C for 30 min and separated on a 0.8% (wt/vol) agarose gel in 1 × TAE (pH 8.0).

Sequencing of AXL3 was performed at The Applied Genomics Core at the University of Alberta. A DNA genomic library was constructed using a Nextera XT library prep kit followed by paired-end sequencing on a MiSeq (Illumina, San Diego, CA) platform using a MiSeq v3 reagent kit.

### 4.8. Bioinformatic Analysis

Quality control analysis was completed using FastQC v0.11.9 [58] and the 1,308,137 paired-end reads were processed using Trimmomatic v.0.38 [59] and the following requirements; removal of Nextera adapter sequences, quality trimming using a four-base sliding window that cuts when the average quality per base drops below 15, removal of the first 20 bases from each read and a minimum read length of 50 bp. 83.74% of both read pairs survived these trimming parameters. SPAdes v.3.12.0 [60] was used to assemble a 47,544 bp contig with 1,998,763 reads mapping to the contig to give a mean coverage of 7516 reads with no gaps or ambiguous sites. The assembly was confirmed with PCR using 13 primer pairs randomly spaced throughout the genome and Sanger sequencing of the PCR products. A primer pair (EndF 5′-CTTGGGTTACAGTGGTGAGC-3′; EndR 5′-AAGGGTGACATCGAGCAGTA-3′) flanking the ends of the contig produced a product with an additional base upon sequencing when compared to the assembled contig, suggesting a complete genome of 47,545 bp in length.

Predicted protein-coding genes were identified using the GLIMMER plugin [61] for Geneious using the Bacteria and Archaea setting, as well as GeneMarkS for phage [62] and Prodigal [63] and annotations to the contig were made using Geneious Prime v2020.0.4 [36]. BLASTn was used to gain information on relatives based on genomic data and putative protein functions were assigned using BLASTp limited to Viruses (taxid:10239), or Bacteria (taxid:2) when Viruses produced no significant hits, on the NCBI non-redundant protein sequence and nucleotide collection databases (update date: 2020/05/19) [64]. Results with E-values greater than 1.00 × 10^−3^ were not recorded and the coding sequences were annotated as hypothetical. Conserved domain searches were performed using CD-Search against the CDD v3.18—55,570 PSSMs database and default options [65] to aid in functional annotation. Lysis protein analysis was performed using TMHMM [66] for transmembrane region identification and LipoP 1.0 [67] for the prediction of lipoproteins. tRNAscan-SE software with the general tRNA model [68] and Aragorn v1.2.36 [69] were used to identify potential tRNA genes. Protein alignments were accomplished using MUSCLE [70].

vConTACT2 (v0.9.16) [35] was used for taxonomic classification against the National Center for Biotechnology Information (NCBI) Prokaryotic Viral RefSeq94-Merged database using Diamond for protein-protein similarity, MCL for protein cluster generation and ClusterONE for viral cluster generation. The resulting network was visualized in Cytoscape (v3.8.0) [71] using an edge-weighted spring-embedded model, which places the genomes sharing more protein clusters closer together.

## Figures and Tables

**Table 1:** Host range analysis of AXL3 on *S. maltophilia* clinical strains.

S. maltophilia Strain	Efficiency of Plating
101	−
102	−
103	−
152	−
155	−
174	−
176	−
213	++++
214	−
217	−
218	−
219	−
230	−
236	−
242	−
249	−
278	−
280	+
282	−
287	−
446	−
667	−
D1585 ^a^	++++
D1571 ^a^	−
D1614 ^a^	−
D1576 ^a^	+
D1568 ^a^	+++
SMDP92	−
ATCC13637	−

−, No sensitivity to phage; +, clearing at 10^10^ pfu/mL undiluted; +++, plaques at 10^−4^; ++++, plaques at 10^−7^. ^a^ Isolates from the Canadian *Burkholderia cepacia* complex Research Referral Repository.

**Table 2:** Genome annotations for AXL3 obtained from BLASTp and CD-Search data. Results below 1.00 × 10^−3^ were not recorded and the function was annotated as hypothetical.

CDS	Coding Region	Strand	Length (AA)	Putative Function	Hit	Species	Coverage (%)	E-Value	Identity (%)	Accession
1	204–806	+	200	hypothetical protein	hypothetical protein	*Burkholderia* sp. SRS-W-2-2016	40	3.00 × 10^−4^	35.56	WP_143752284.1
2	806–2368	+	520	large terminase	terminase large subunit	*Microcystic* phage Me-ZS1	95	0	59.96	AZF88145.1
3	2379–3887	+	502	portal protein	putative portal protein	Prokaryotic dsDNA virus sp.	97	2.00 × 10^−141^	47.7	QDP56576.1
4	3891–4952	+	353	head morphogenesis protein	hypothetical protein	uncultured *Caudovirales* phage	98	1.00 × 10^−91^	44.17	ASN68636.1
5	4998–5156	+	52	hypothetical protein	-					
6	5177–5911	+	244	scaffold protein	scaffold protein	*Pseudomonas* phage vB_PaeS_SCH_Ab26	99	6.00 × 10^−41^	41.3	YP_009044344.1
7	5946–6962	+	338	capsid protein	capsid protein	*Salmonella* phage PMBT28	98	8.00 × 10^−124^	51.46	AUZ95497.1
8	7041–7340	+	99	hypothetical protein	-					
9	7347–7871	+	174	structural protein	structural protein	*Achromobacter* phage vB_Ade_ART	99	1.00 × 10^−32^	45.56	AYD82587.1
10	7876–8259	+	127	head-tail joining protein	hypothetical protein	*Pseudomonas* phage PaSz-4	100	4.00 × 10^−14^	32.56	QAX99460.1
11	8256–8687	+	143	structural protein	putative structural protein	*Pseudomonas* phage PAE1	94	3.00 × 10^−33^	41.55	YP_009215709.1
12	8692–10,245	+	517	major tail structural protein	major tail structural protein	*Pseudomonas* phage NP1	98	0	65.36	YP_009285827.1
13	10,272–10,691	+	139	structural protein	putative structural protein	*Pseudomonas* phage NP1	100	4.00 × 10^−56^	57.55	YP_009285828.1
14	10,712–11,071	+	119	hypothetical protein	hypothetical protein	*Pseudomonas* phage LKO4	73	9.00 × 10^−27^	54.02	YP_009601866.1
15	11,068–11,496	+	142	hypothetical protein	hypothetical protein	*Bordetella* phage FP1	98	2.00 × 10^−37^	50	YP_009794086.1
16	11,501–14,341	+	946	tape measure protein	hypothetical protein	*Pseudomonas* virus M6	98	0	47.08	YP_001294539.1
17	14,352–15,296	+	314	tail assembly protein	tail assembly protein	*Xylella* phage Salvo	99	6.00 × 10^−133^	58.72	YP_009639180.1
18	15,296–16,279	+	327	hypothetical protein	hypothetical protein	*Stenotrophomonas* phage vB_SmaS-DLP_1	98	1.00 × 10^−72^	42.04	AKI28800.1
19	16,282–18,018	+	578	hypothetical protein	hypothetical protein	*Pseudomonas* phage vB_PaeS_C1	91	2.00 × 10^−115^	36.84	AVJ48097.1
20	18,015–18,854	+	279	hypothetical protein	hypothetical protein	*Burkholderia* phage vB_BceS_KL1	99	3.00 × 10^−80^	45.68	YP_006560776.1
21	18,858–21,968	+	1036	central tail hub	tail protein	*Burkholderia* phage vB_BceS_KL1	77	0	46.73	YP_006560777.1
22	21,965–22,138	+	57	hypothetical protein	-					
23	22,135–22,584	+	149	endolysin	endolysin	*Xanthomonas* phage Xp15	98	2.00 × 10^−53^	53.9	YP_239293.1
24	22,590–23,021	+	143	hypothetical protein	-					
25	23,018–23,323	+	101	hypothetical protein	hypothetical protein	*Sinobacteraceae* bacterium	92	1.00 × 10^−9^	40	TXH02718.1
26	23,579–23,941	+	120	hypothetical protein	-					
27	24,050–24,571	−	173	dCMP deaminase	dCMP deaminase	uncultured Mediterranean phage uvMED	91	6.00 × 10^−43^	47.17	YP_009778145.1
28	24,942–25,751	−	269	thymidylate synthase	thymidylate synthase	*Pelagibacter* phage HTVC200P	87	6.00 × 10^−65^	44.49	AXH71582.1
29	25,732–26,715	−	327	thymidylate kinase	thymidylate kinase	*Caudovirales* sp. ctOwN3	93	3.00 × 10^−33^	33.98	QGH72154.1
30	26,696–27,250	−	184	nucleoside triphosphate pyrophosphohydrolase	NTP-Ppase	*Caudovirales* sp. ctOwN3	69	5.00 × 10^−32^	46.21	QGH72159.1
31	27,260–28,084	−	274	methyltransferase	methyltransferase domain-containing protein	*Nitrospira cf. moscoviensis* SBR1015	94	5.00 × 10^−133^	73.08	WP_087475488.1
32	28,162–28,761	−	199	hypothetical protein	hypothetical protein	*Nitrospira cf. moscoviensis* SBR1015	74	3.00 × 10^−26^	44.13	WP_087475489.1
33	29,100–29,273	−	57	hypothetical protein	-					
34	29,273–29,920	−	215	hypothetical protein	-					
35	29,997–30,089	−	30	hypothetical protein	-					
36	30,100–30,375	−	91	hypothetical protein	hypothetical protein	*Xanthomonas* phage Xoo-sp2	93	4.00 × 10^−20^	48.24	ANT45253.1
37	30,379–30,588	−	69	hypothetical protein	-					
38	30,700–32,256	−	518	DNA helicase	hypothetical protein	Prokaryotic dsDNA virus sp.	91	4.00 × 10^−123^	44.49	QDP55885.1
39	32,463–32,690	−	75	hypothetical protein	-					
40	32,678–33,532	−	284	RecB family exonuclease/Cas4	putative RecB family exonuclease	*Campylobacter* phage vB_CjeM_Los1	84	1.00 × 10^−10^	27.31	YP_009597155.1
41	33,532–35,457	−	641	DNA polymerase I	DNA polymerase A	*Vibrio* phage VpKK5	71	1.00 × 10^−29^	27.62	YP_009126593.1
42	35,454–35,672	−	72	hypothetical protein	hypothetical protein	*Sandarakinorhabdus limnophila*	72	4.00 × 10^−5^	46.15	WP_022681046.1
43	35,750–36,652	−	300	hypothetical protein	hypothetical protein	*Pseudomonas* phage KPP25	72	4.00 × 10^−12^	24.55	YP_009030602.1
44	36,720–37,232	−	170	hypothetical protein	hypothetical protein	*Nitrospira cf. moscoviensis* SBR1015	76	9.00 × 10^−35^	52.31	WP_087475496.1
45	37,232–37,702	−	156	hypothetical protein	-					
46	37,789–38,694	−	301	DNA ligase	DNA ligase	*Xanthomonas* phage Xoo-sp2	99	3.00 × 10^−81^	45	ANT45243.1
47	38,691–39,158	−	155	hypothetical protein	hypothetical protein	*Microcystic* phage Me-ZS1	92	1.00 × 10^−25^	40.28	AZF88158.1
48	39,155–39,403	−	82	hypothetical protein	hypothetical protein	*Cupriavidus* sp. UYMSc13B	100	3.00 × 10^−9^	37.35	RWA55322.1
49	39,405–39,674	−	89	hypothetical protein	-					
50	39,667–40,515	−	282	thymidylate synthase complementing protein	thimidilate synthase	*Caulobacter* phage Seuss	98	3.00 × 10^−66^	43.77	YP_009785564.1
51	40,505–40,747	−	80	hypothetical protein	-					
52	40,816–41,031	−	71	hypothetical protein	-					
53	41,028–41,546	−	172	hypothetical protein	hypothetical protein	*Mycobacterium* phage OkiRoe	56	4.00 × 10^−9^	36.61	YP_009043654.1
54	41,548–41,745	−	65	hypothetical protein	-					
55	41,742–42,221	−	159	DUF1643 containing protein	hypothetical protein	*Pseudomonas* phage Epa33	95	2.00 × 10^−47^	53.85	QIQ65784.1
56	42,218–42,520	−	100	hypothetical protein	hypothetical protein	*Xylella* phage Sano	99	3.00 × 10^−16^	42.16	YP_009639092.1
57	42,517–42,801	−	94	hypothetical protein	-					
58	42,830–42,973	−	47	hypothetical protein	-					
59	42,970–43,182	−	70	hypothetical protein	-					
60	43,179–43,514	−	111	DnaJ domain-containing protein	DnaJ domain-containing protein	*Pseudoalteromonas* sp. FUC4	53	2.00 × 10^−5^	38.33	WP_149594670.1
61	43,511–43,693	−	60	hypothetical protein	-					
62	43,756–44,055	−	99	Glu-rich hypothetical protein	-					
63	44,148–44,369	−	73	hypothetical protein	-					
64	44,463–46,577	−	704	Primase	putative primase	*Stenotrophomonas* phage S1	33	4.00 × 10^−28^	32.91	YP_002321451.1
65	46,876–47,385	−	169	hypothetical protein	-					

**Table 3:** The conserved domains found in the 65 gene products of AXL3.

Gp	Hit Type	PSSM-ID	Interval	E-Value	Accession	Short Name	Superfamily
2	Superfamily	392065	31–274	1.96 × 10^−3^	cl29365	Terminase_6 superfamily	-
3	Specific	372539	261–406	6.28 × 10^−34^	pfam13264	DUF4055	cl16196
4	Superfamily	385666	186–261	3.11 × 10^−12^	cl10072	Phage_Mu_F superfamily	-
4	Superfamily	225244	30–263	2.65 × 10^−5^	cl26983	COG2369 superfamily	-
6	Superfamily	374274	41–106	2.85 × 10^−3^	cl25765	G_path_suppress superfamily	-
7	Superfamily	391678	156–250	9.22 × 10^−3^	cl27082	Phage_capsid superfamily	-
11	Superfamily	372633	16–94	1.13 × 10^−4^	cl16304	DUF4128 superfamily	-
16	Specific	131723	128–202	7.49 × 10^−19^	TIGR02675	tap_meas_nterm	cl31236
16	Specific	227606	114–682	4.31 × 10^−13^	COG5281	COG5281	cl34971
20	Superfamily	378160	211–271	7.98 × 10^−11^	cl10710	Phage_BR0599 superfamily	-
21	Specific	379255	278–448	2.40 × 10^−13^	pfam13550	Phage-tail_3	cl38419
21	Superfamily	389952	874–1024	9.63 × 10^−4^	cl22861	LamG superfamily	-
23	Specific	350620	9–147	4.62 × 10^−29^	cd14845	L-Ala-D-Glu_peptidase_like	cl38918
27	Superfamily	381914	6–159	1.31 × 10^−36^	cl00269	cytidine_deaminase-like superfamily	-
28	Superfamily	388507	30–219	1.84 × 10^−31^	cl19097	TS_Pyrimidine_HMase superfamily	-
30	Specific	212137	34–122	1.32 × 10^−21^	cd11530	NTP-Ppase_DR2231_like	cl16941
31	Superfamily	225139	52–192	1.16 × 10^−10^	cl34437	Cfa superfamily	-
38	Specific	223627	7–509	7.90 × 10^−32^	COG0553	HepA	cl33945

**Table 4:** Strains and plasmids used in this study.

Bacterial Strain	Genotype or Description	Source
D1585	Wildtype, AXL3-sensitive	CBCCRRR *
D1585 Δ*pilA*	Clean deletion of *pilA* in D1585	[26]
D1585 Δ*pilT*	Clean deletion of *pilT* in D1585	[19]
D1571	Wildtype, AXL3-resistant	CBCCRRR *
**Plasmids**		
pBBR1MCS	Broad-host range cloning vector, Cm^R^	[54]
pD1585*pilA*	pBBR1MCS carrying D1585 *pilA*, Cm^R^	[26]
pD1585*pilT*	pBBR1MCS carrying D1585 *pilT*, Cm^R^	[19]

* Canadian *Burkholderia cepacia* complex Research Referral Repository.

## Supplementary Materials

Supplementary materials can be found at https://www.mdpi.com/1422-0067/21/17/6338/s1.
