# Supplementary material for: Isolation and Characterization of the Novel Bacteriophage AXL3 against Stenotrophomonas maltophilia
Source: Int J Mol Sci. 2020 Sep 1;21(17):6338. doi: 10.3390/ijms21176338 (PMC7504290; doi:10.3390/ijms21176338)
Supplement: Supplementary file 1 [file ijms-21-06338-s001.pdf]

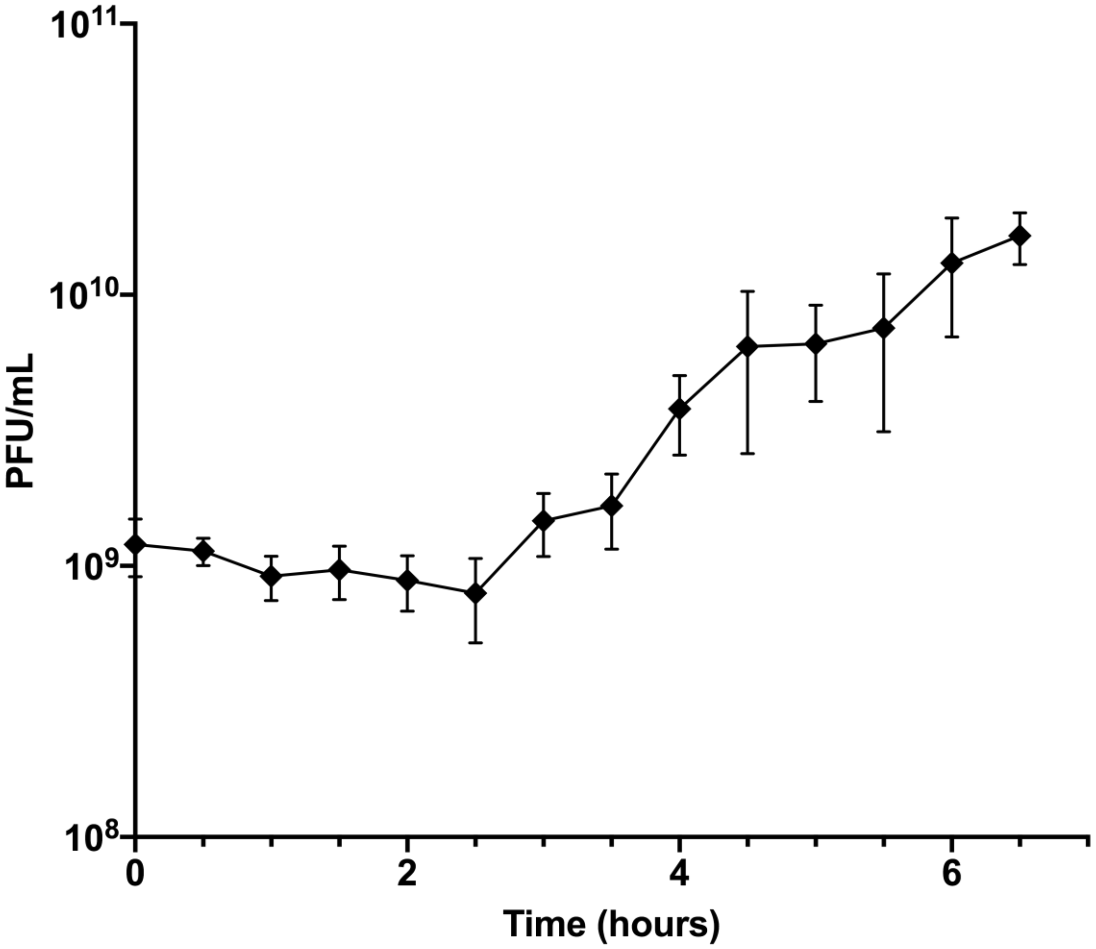

Figure S1: AXL3 one-step growth curve on *S. maltophilia* strain D1585. Subcultured D1585 was grown to OD_{600} of 0.2 in LB at 30°C. AXL3 lysate was added at an MOI of ~3, adsorbed for 5 min, followed by incubation at 30°C with aeration at 225 RPM. Samples were taken at 30 min intervals, serially diluted, and 5 μL of each dilution was spotted on soft agar overlays containing D1585. Data from four replicates shown. Phage AXL3 exhibits a latent period of 2.5 hours, and a burst size of 38 virions per cell at 6.5 hours.

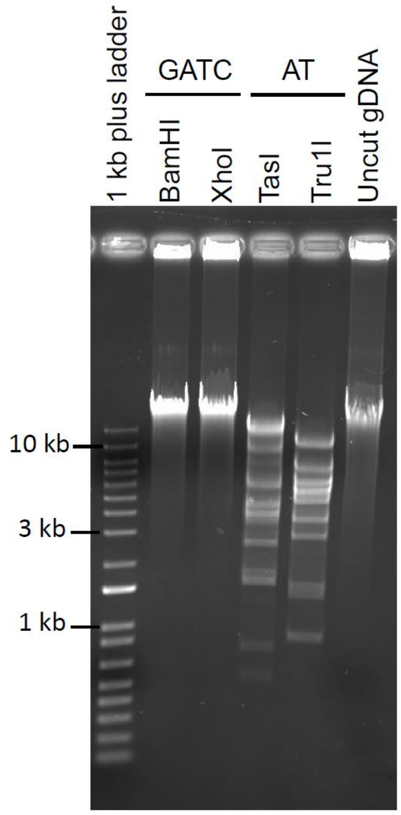

Figure S2: Restriction digests of AXL3 gDNA showing base modification. Agarose gel electrophoresis of AXL3 gDNA following incubation with *BamHI* and *XhoI* enzymes containing GATC bases in their recognition sites compared with *TasI* and *Tru1I* enzymes containing only AT bases in their recognition sites.

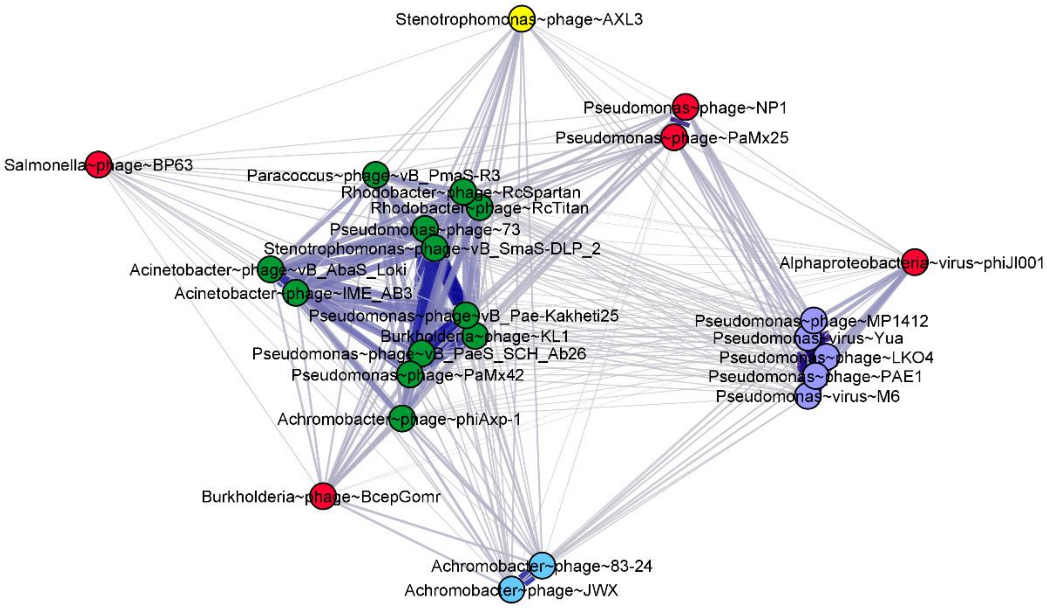

Figure S3: Network representation of AXL3 phylogeny. Analysis with vConTACT2 [36] identifies AXL3 as an outlier genome weakly connected to three phage clusters. This network comprises 24 out of 2,617 National Center for Biotechnology Information (NCBI) RefSeq phage genomes with connections to the AXL3 genome (yellow), each represented by a node (circles). Each colour

represents a viral cluster, with red and yellow indicating outliers. An edge (line) represents a connection between two nodes (genomes) based on the number of shared protein clusters, with darker and thicker edges indicating increased significance. Visualization of the network was performed using Cytoscape [71].
